# Process vs. Processor Accounts of Stage Models: A Cautionary Tale. Commentary: Seeing changes: How familiarity alters our perception of change

**DOI:** 10.3389/fpsyg.2016.00719

**Published:** 2016-05-12

**Authors:** Luke Kersten

**Affiliations:** Department of Cognitive Science, Institute of Cognitive Science, Carleton UniversityOttawa, ON, Canada

**Keywords:** stage models, processing devices, process modules, additive factors method, temporal stages

Sternberg (2001) defines *processing modules* as distinct parts that are separately modifiable—a process is separately modifiable when each of its modules can be modified without effect on other modules. Sequentially arranged process modules are “stages” and explanations aimed at decomposing complex processes into stages are “stage models.” In this commentary, I look at one issue that arises for Tovey and Herdman's (2014) stage model of change perception. I argue that Tovey and Herdman's introduction of a “gating mechanism” complicates interpretation of their model as a stage model. I use Tovey and Herdman's model as a cautionary example for interpreting stage models generally.

One aspect of stage models that is sometimes underappreciated is that the process modules revealed by techniques such as the additive factors method are functions operating over epochs of time rather than functional components operating in space. Process modules are *actions* rather than *actors* (see, e.g., Townsend and Ashby, 1983; Townsend and Nozawa, 1995; Sternberg, 2001). This feature of stage models is important, as it is sometimes tempting to assume that information flow between process modules is also information flow between *processing devices*.

One issue with this way of thinking is that it can produce misleading accounts of how process modules relate to processing devices. There are many processor types that can carry out the same set of processing stages. Several possible relations exist for even a three-stage process, for example. A separate processor might carry out each process, the same processor might carry out every process, or there be might some complex combination of the two, where one processor carries out two processes and another processor carries out one process. To avoid confusion, stage models should avoid discussion of processor devices where possible.

The slide between process and processor descriptions of stage models is easy to make. Consider, for example, Tovey and Herdman (2014). Tovey and Herdman investigated the effects of familiarity on change perception using a 2 × 5 × 2 factorial design. They examined the effects of orientation (upright vs. inverted, set size (4, 7, 10, 13, 16) and change size (Small vs. Large) across four different experiments. In line with Rensink (2000a,b, 2002, 2005), they suggested that change perception is divided into three process modules: a pre-processing, feature extraction, and identification stage. Tovey and Herdman (2014, p. 232) proposed that an interaction between change size and orientation and change size and stimulus quality suggested that change size exerted an effect not only at the feature extraction stage but also at the identification stage of change perception. This prompted them to propose a “gating mechanism.” They claimed that when changes in size are detected they are either passed on to the feature-extraction stage (assuming they are large) or retained and verified at the identification stage (assuming they are small). Figure 1 provides an illustration.

**Figure 1:**
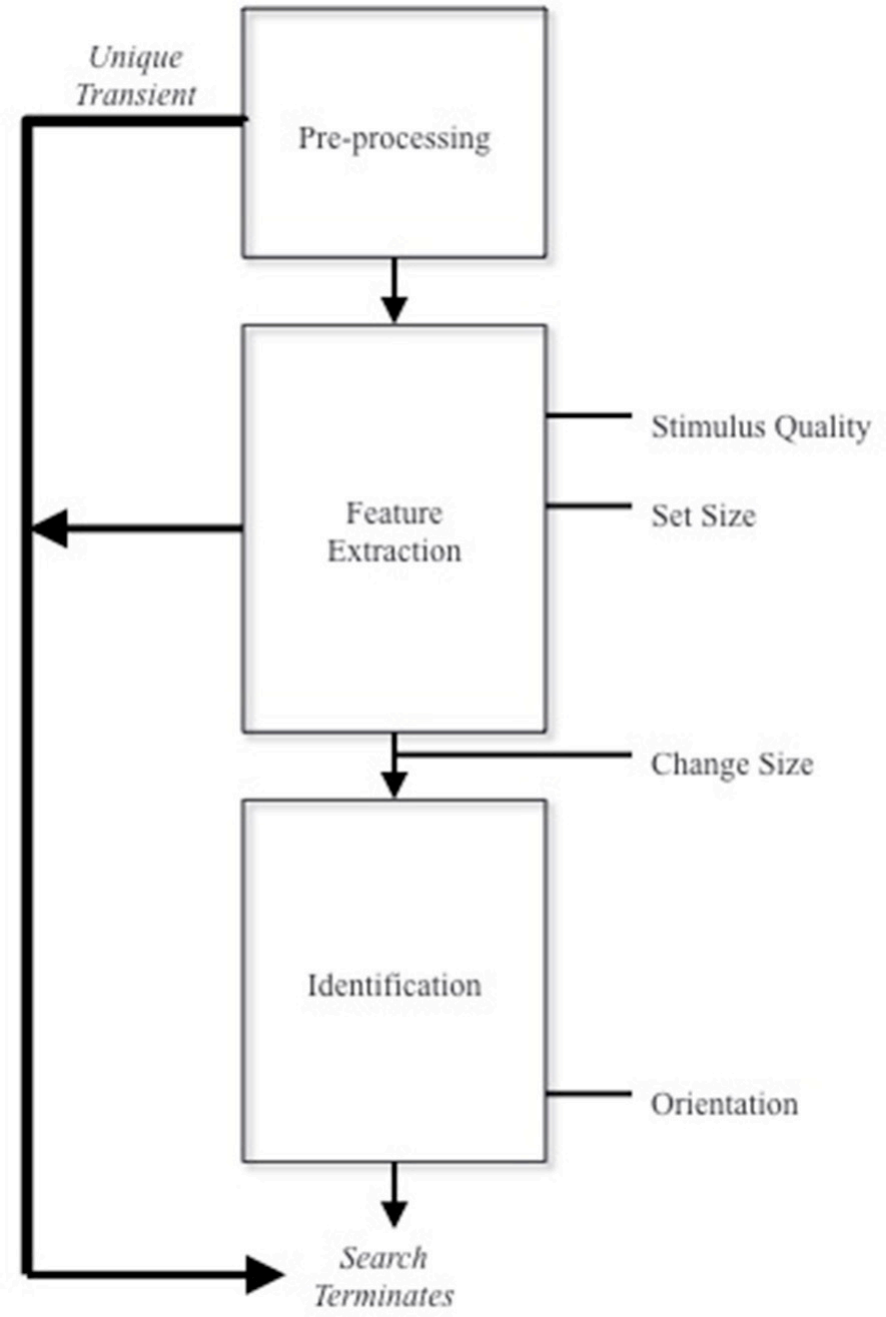
**Tovey and Herdman's stage model**.

Introduction of a gating mechanism, however, complicates interpretation of Tovey and Herdman's model as a serial stage model. This is because it introduces a functional property into the model. Process modules, at least traditionally understood, are not the sorts of structures that possess functional properties (e.g., the ability to channel or redirect information). They are operations carried out over successive periods of time, not physical structures with causal powers. The processor devices that carry out process modules might have functional properties, but the processing modules themselves, at least as informed by techniques such as the additive factors method, are neutral with respect to such questions (see Sternberg, 2001).

Tovey and Herdman have unduly inferred processor properties on top of process modules. Though Tovey and Herdman's model may be correct, inclusion of functional properties complicates interpretation of the model as a serial stage model. It is not that the results of Tovey and Herdman are suspect, but their interpretation of the model.

Another reason to think that Tovey and Herdman's model has slid into discussion of processing devices is the diagram provided by the model. Replicated in Figure 1, Tovey and Herdman's model places change size outside of any processing stage, residing after feature extraction but before identification. This seems to change the structure of the diagram from a flowchart to a circuit diagram, as the arrows no longer represent a succession in time of a series of processes but the flow of information from one processor to another. The problem, as Sternberg (2001) points out, is that since process modules are events in time they need to be strung together end to end, as in a flowchart. When represented as a circuit diagram—that is, as describing how processing devices are connected—stage models can misleading suggest that the process modules are, in fact, also processing devices arranged in a serial sequence; an interpretation, as mentioned, that fails to acknowledge the variety of possible relationships that might obtain between process modules and processing devices.

Tovey and Herdman might, in response, point out that change size is conceived of as factor, and that therefore its inclusion outside of the stage merely denotes where its effect is felt. Though, I am sensitive to this interpretation, my worry is that it does not satisfactorily resolve the issue. The gating mechanism is still conceived of as the change size. It therefore denotes the redirection of information from one stage to another, not only how change size influences time duration. If the model represented the effect of change size, it would have to effect the period of time as represented by the box, not the passage or succession of time as represented by the arrows.

The slide between process/processor interpretations of stage models is subtle but important. In using Tovey and Herdman's model as a representative example, I have tried to highlight the cognizance required to avoid interpretative and conceptual confusion within discussions of stage models. One way to see the current commentary, then, is not so much as a criticism but as a cautionary tale for future research.

## Author contributions

The author confirms being the sole contributor of this work and approved it for publication.

### Conflict of interest statement

The author declares that the research was conducted in the absence of any commercial or financial relationships that could be construed as a potential conflict of interest.
